# The mediating effect of knowledge sharing in the relationship between factors affecting knowledge sharing and reflective thinking: the case of English literature students during the COVID-19 crisis

**DOI:** 10.1186/s41039-022-00200-3

**Published:** 2022-06-11

**Authors:** Majid Farahian, Farshad Parhamnia, Nasser Maleki

**Affiliations:** 1 Department of ELT, Kermanshah Branch, Islamic Azad University, Kermanshah, Iran; 2 Department of Knowledge and Information Science, Kermanshah Branch, Islamic Azad University, Kermanshah, Iran; 3 Department of English Language and Literature, Razi University, Kermanshah, Iran

**Keywords:** Individual factors, Classroom factors, Technological factors, Knowledge sharing, Reflective thinking, English literature students

## Abstract

The COVID-19 pandemic has had serious implications on educational systems worldwide and, hence, online courses have been organized. It is expected that the use of online learning in higher education promote knowledge sharing among students and the sharing of knowledge result in the improvement of reflective thinking among them. As such, we examined the knowledge sharing behavior among the undergraduate students in online learning English literature courses, the student’ perceptions towards reflective thinking, the relationship between the students’ knowledge sharing and reflective thinking, and finally, we tested a structural model of factors affecting knowledge sharing components, knowledge sharing, and reflective thinking. The data were collected through two surveys of 104 Iranian English literature students. A Pearson’s correlation coefficient and path analysis were used to analyze the data and to generate a model. The results showed that the students’ online knowledge sharing behavior and their perceptions towards reflective thinking are at unacceptable levels. Furthermore, a significant relationship was found between factors affecting knowledge sharing with the students’ knowledge sharing behavior, and between knowledge sharing and reflective thinking. The results also confirmed the mediator role of knowledge sharing and supported the hypothesized model of the relationships among the variables. Pedagogical implications of the study are finally discussed.

## Introduction

Over the last few decades, EFL has faced many challenges, therefore, creating fundamental innovations in the curriculum and overcoming the challenges has always been the goal of researchers and scholars (Sun & Chen, 2016). In recent years, the nature of teaching and learning English has changed so much that by placing text, audio, and video on the World Wide Web, there is a unique opportunity to use these multimedia systems to teach and learn foreign languages. Due to the advantages of this way of learning, the importance and application of virtual teaching and learning method is increasing every day (Allen & Seaman, 2013; Chou, 2010; Li & Akins, 2004). The optimal use of technology in language teaching can provide benefits for both students and teachers, such as creating an environment in which more students can participate in classroom activities. It also can encourage more engaged individual learning and promote students’ motivation as the result of the optimal use of technology (Abou El-Seoud, et. al, 2014; Rajaee Harandi, 2015; Rovai et al., 2007).

Online education has created new opportunities in teaching and learning and has made it possible for any person, at any time and place to get involved in the teaching learning process (Britt, 2006; Che Musa et al., 2012; Hubbard, 2008; Khan, 2004). Using online learning, teachers and practitioners transmit the educational content to learners through course management softwares, multimedia resources, the Internet, and video conferencing. It should be noted that online learning has been used interchangeably with other similar terms such as virtual learning, computer-based learning, and e-learning; however, since the term online education covers a much broader range of educational services (Paulsen, 2002) the term online learning is used in this paper to reduce misconceptions about the overlapping terms.

With the outbreak of the COVID-19 pandemic and the closure of educational institutions, a deeper look at online learning seems to be more essential than ever since one of the most important factors in the use and prevalence of this type of education is the COVID-19 disease which has affected educational settings such as schools and universities all over the world, and Iran is no exception. Although the social media has been extensively used throughout the country and the new technologies have been integrated into educational system, the new e-learning opportunity has turned to be ‘emergency e-learning’. According to Hodges et al. (2020) emergency e-learning is “the temporary shift of instructional delivery to an alternate delivery mode due to crisis circumstances” (p. 6). The difficulty of managing this crisis, especially with the special conditions of Iran being under heavy economic sanctions and the lack of sufficient experience in this regard (Nikdel Teymori & Fardin, 2020) from one hand, and the condition imposed by emergency e-learning on the other hand, provided the field for creativity and various innovations. These valuable experiences can turn to valuable assets to the health care as well as the educational system of the country through genuine and timely decisions. Hence, universities and educational institutions began to launch virtual education systems and tried to change classes from face-to-face to virtual courses. One of the systems, the Learning Management System (LMS) as a platform in online learning (Ebadi et al., 2020) has been used in state universities throughout the country. In its very basic form, the platform was introduced to the context of Iranian educational system in 1990 (Mahmoudi-Dehaki et al., 2021).These universities have set up the LMS platforms in order to integrate and improve the quality level of distance and online education. In order to facilitate educational and student affairs, they have updated and improved its electronic infrastructure and systems with a large number of students and educational units scattered throughout the country and in different cities. Despite the benefits, such as accessibility to the course from anywhere at any time, asynchronous discussions with teachers and classmates, immediate feedback on tests, and so on (Ahmady et al., 2020), based on the researchers’ experience, it seems that due to the nature of online learning, knowledge sharing among students and teachers is to some degrees different from the one in face-to-face courses since online learning may affect teacher to students and student to student interaction. Perhaps, the quality and type of such interactions affect students’ reflective thinking and mental development (Alblehai & Umar, 2016; Vygotsky, 1978).

The use of virtual learning courses does not necessarily guarantee the success of the educational system (Sanchez, 2011). Meanwhile, considering that virtual education is a new development in the country’s educational system and that universities are under the pressure of ‘emergency e-learning’ the literature on the issue of knowledge sharing through online systems and its possible relationship with reflective thinking as an important outcome of education is poor although both concepts are separately well-discussed in the literature. The study of knowledge sharing in the pandemic seems to be crucial since the effectiveness of the courses is contingent on teachers’ and students’ capacity to, elaborate, collect, share, and, transfer knowledge (Viner et al., 2020). Additionally, even though the relationship between collaborative learning and higher-order thinking and reflective thinking has been fully discussed in the literature, both conceptually and empirically, (e.g., Lopes et al., 2018; Qin, 1992; Susanti et al., 2020; Yaacob et al., 2021) little work has examined how knowledge sharing may impact students’ higher-order and reflective thinking (e.g., Alblehai & Umar, 2016; Ricci, 2009). Based on the literature, it can be speculated that scaffolding triggered in students’ interaction can encourage students’ cognitive development in general and reflective thinking in particular. This gains support from Vygotsky’s ZPD theory which posits that the appropriate assistance one received from a more competent peer leads to the development of his/her higher order thinking skills (Vygotsky, 1978).

Accordingly, based on the gap in the literature we examined the knowledge sharing behavior among the undergraduate students in online learning English literature courses, the student’ perceptions towards reflective thinking, the relationship between the students’ knowledge sharing and reflective thinking, and finally, we tested a structural model of factors affecting knowledge sharing components, knowledge sharing, and reflective thinking.

### Literature review

In the following section, an overview of important concept and variables are presented.

### Knowledge sharing

Knowledge management is “the process of collecting, managing and sharing … knowledge in an organization” (Bhojarajo, 2005, p. 37). Members of an organization use knowledge management to “create, share, and apply knowledge to achieve their strategic and operational goals” (North & Babakhanlu, 2016, p. 211). Knowledge sharing, as an important subset of knowledge management, “is the acquisition, organization, reuse and transfer of experience-based knowledge and making that knowledge available to others” (Lin, 2006, p. 27). Although the concepts knowledge management and knowledge sharing were first introduced in business organizations, they are crucial for knowledge management practices in knowledge-based organizations like universities (Cheng et al., 2009) since universities revolve around several fundamental knowledge processes, namely knowledge creation; knowledge dissemination and learning (Trifonova & Ronchetti, 2006). As such the idea of knowledge sharing has recently been widely used in the related literature (e.g., Akosile & Olatokun, 2019; Bibi &Ali, 2017; Fullwood et al., 2018; Tan, 2015).

It is through knowledge sharing that experience-based knowledge is made available and transmitted to other members (Lin, 2006). Efficient knowledge sharing promotes an organization's chances of survival (Arogot et al., 2003). As such, it seems that online networks which are flourishing and have gained popularity may pave the way for such a purpose, although scant attention has been paid to the nature of interaction in online courses from teachers’ and students’ views (Blaine, 2019). Nowadays, multiple technologies have been used to support creating, organizing access and using the intellectual assets (Nassuora, 2011). Utilization of information technology has a fundamental role in knowledge sharing (Davenport & Prusak, 2000) and the growth of knowledge management has been closely linked to information technology (Chumer et al., 2000). However, the key challenge is how in online social network communities, knowledge sharing can be achieved effectively (Charband & Jafari Navimipour, 2016; Nassuora, 2011).

Regarding knowledge management in virtual courses, Heidari (2020) addressed the need for knowledge sharing in online courses in the crisis of the COVID-19 outbreak. With respect to the quality of e-learning compared to face-to-face classes, Rasouli et al., (2016) consider lack of interaction in virtual courses and improper sharing of knowledge among participants in the classroom as an important barrier in online courses. In the same line, Brown (2000) concluded that learning is facilitated through virtual learning, but the learners in his study stated that they do not have an effective relationship with other group members and teachers. In the study, he pointed to some factors namely, teacher perseverance, organization, high commitment to learner interaction, and knowledge of technology as factors that can promote e-learning. Pan et al. (2001) have also shown that for successful knowledge sharing, the successful creation of a knowledge-sharing environment is essential. In this regard, Majid et al., (2014) who explored students’ preference for participation in face-to-face and online discussions reported that for a majority of the students, knowledge sharing took place in physical face-to-face discussions rather than online courses. Similarly, Gillies (2008) who explored student teacher interaction in a synchronous learning of video conferencing reported that teachers’ monotonous instruction, students’ lack of eye contact with the teacher, and communication breakdowns resulted in a low level of interaction.

### Reflective thinking

Another important concept discussed in the present study which may be affected by students’ knowledge sharing is higher-order thinking in general and reflective thinking in particular (Alblehai & Umar, 2016). Although there is no agreement upon a unified definition for the term reflection, the pioneers in reflective thinking consider it as a special type of problem-solving that entails linking and synthesis of ideas (Hatton & Smith, 1995). Reflective thinking is related to thinking, analyzing and making judgment about the events that have happened and those which are happening (Norton, 1997). It has been argued that students who analyze ideas and have a critical stance towards events can see and understand the content of the lessons on a more lasting and deeper level (Paul & Elder, 2008).

As to the stages of reflection, various theoretical frameworks have been suggested for reflection (e.g. Gibbs, 1988; Kolb, 1984; Mezirow, 1991). Gibbs (1988) as a widely known framework draws on Kolb’s (1984) cycle and suggests a more detailed process for exploring and analyzing the situation. In the framework, Gibbs lists the basic stages of reflection which seem to be globally inclusive. In other words, they seem to be at work across a wide range of activities from general to academic. In addition, it has clear and elaborated stages illustrates learners’ opportunity to reflect on their experience, and to understand what they did well and what they could do in the future (Hussein, 2018). There are six stages in Gibbs’ reflective cycle including description, feelings, evaluation, analysis, conclusion, and action plan. Description refers to the factual description of the event. Feelings has to do with one’s emotions during and after the event. At the third stage of the cycle, one gets involved in an objective evaluation of the situation and he/she takes into consideration of how well things went. Finally, at the last stage, one considers the lessons he/she has learned from the events and whether he/she could have responded in a different way in the event.

In the Iranian educational context, the customary teaching practices are mostly memorization-driven and teacher-centered while students are rarely given the opportunity or guidance to evaluate let alone question the knowledge transmitted to them (Avarzamani & Farahian, 2019; Enayat et al., 2015). Equally, teachers and university professors receive little training regarding higher-order thinking. Unlike the status quo inclined towards a reproduction-oriented curriculum, there has been an ardent attempt by the Ministry of Education in Iran to follow the educational reform in Western and East Asian educational context introducing reflective thinking into curricula in different levels of education. While such an attempt is still far away from the ideal condition, it seems that this new move has the potential to influence teachers in two different ways. There is a hope that such a movement can raise teachers’ awareness regarding higher-order thinking and as the result they teach reflectively. It can also help teachers reverse their learning habits by helping them develop higher-order thinking skills and nurture inclination towards reflectivity. It seems that literature has an indisputable role in this regard (Dickson, 1991; Van, 2009).

### English literature courses and reflective thinking

As Tung and Chang (2009) argue, literature reading requires students to recall, retrieve and reflect on their prior experiences or memories to construct meanings of the text. While they are doing so, they need to demonstrate skills and abilities used in higher-order thinking such as exercising interpretation, evaluation, analysis, synthesis, argumentation, inferencing, and reasoning. In addition, as Tung and Chang further argue, literature provides readers with instances of real-world context in which readers need to delve into their own personal experiences to construct meanings. However, despite the nature of literature which derives students towards higher-order thinking, as Shukri and Mukundan (2015) note in their review of literature, teaching literature alone does not ensure students’ promotion of higher-order thinking. In this regard, Khatib and Nima (2013, P.105) explain that for such a purpose different conditions should be met among which promoting interaction in the classroom seems to be of a high priority. As they explain, lots of opportunities should be created “for peer interaction around reasoning”. This includes interactions in which literature students “reason together, discuss reasoning with one another, and evaluate reasoning together”. This may help students, as Katib and Nima further put it, “by anchoring it in meaningful interpersonal interactions” which may be realized through knowledge sharing among students. In the same vein, Shukri and Mukundan (2015) explain that literature does not lead to the improvement of higher-order thinking unless students are engaged in the classroom practice.

### Individual, classroom, technological factors and knowledge sharing

It has been speculated that individual factors affect knowledge sharing since “the heart of any effective change is the people change” (p.2). The influence of various individual factors such as personality and attitude (Awad & Ghaziri, 2004), motivations, trust and care (Van den Brink, 2003), self-efficacy (Lin, 2007) on knowledge sharing has been supported by research. Wangpipatwong (2009) also reported that degree of competition, has a significant impact on knowledge sharing. Nisar ul Haq and Haque (2018) found that trust, and attitude among other factors are the key factors in order to improve knowledge students’ knowledge sharing. Jantavongso and Nuansomsri (2019) in their study also concluded that "intention to share", "technological", "individual", and "classroom" factors affected knowledge sharing among ICT undergraduate students.

A large number of studies have found that there is a beneficial association between organizational variables and employee knowledge sharing (Liu & Liu, 2011; Lu et al., 2006; Razmerita et al., 2016). Although the present study focuses on student knowledge sharing, the basic principles concerning organizational elements might be applicable to the classroom environment. As to the relationship between classroom factors and knowledge sharing, several studies have found evidence of a positive correlation between various aspects of the classroom environment, such as peer relationships, the teacher's grading system, the level of competition in the classroom, and students' knowledge sharing behavior (Nuansomsri & Jantavongso, 2016; Riege, 2005). Instructor support, among the various characteristics of classroom dynamics, may play a key role in the process of knowledge transfer. In this regard, Nuansomsri and Jantavongso (2016) reported that there is a positive relationship between instructor support and student knowledge sharing.

Technology has an important role in knowledge management. Although technology is not the center of knowledge management, it plays a critical role as an enabler in increasing the level of knowledge sharing among individuals (Andersson, 2000). It promotes and facilitates the process of knowledge sharing both intra and inter organizations (Gurteen, 1999). The functions of coordination and communication within and inter organizations can be influenced by information technology (Fountain, 2001). The role of information technology in knowledge sharing has been studied by communication theorists (Binz-Scharf, 2008). For example, McDermott (1999) encourages organizations to develop new ways of sharing knowledge among the individuals like using electronic networks to share knowledge within the entire organizations and storing documents in a common knowledge base. Riege (2005) cited in Ismail and Yusof (2010, p.242) reported that there are seven technological factors that are barriers to sharing knowledge. These factors include lack of information technology process and system integration, lack of internal and external technology support, unrealistic expectation what technology can do and cannot do, Mismatch between technological needs, systems integration and information technology processes, reluctant to use information technology because of not familiar to, Lack of training to get use to new information technology systems and processes, and lack of communication and usage of new system advantages compared to current system.

### Knowledge sharing and reflective thinking

Although both knowledge sharing and reflective thinking are well discussed in the literature, there is dearth of research on the relationship between knowledge sharing and reflective thinking. There are scholars (Alblehai & Umar, 2016; Lipman, 1991; Vygotsky, 1978) who consider social interaction and scaffolding as affecting students’ higher-order thinking and mental development. It has also been demonstrated that web-based technology develops problem-solving skills (Zarei et al., 2019). Rahmi andet al. (2020) have also reported that Scaffolding, that may be gained via knowledge sharing can encourage and trigger students’ reflective thinking.

### The present study

Various studies have been conducted on the importance of e-learning during the outbreak of COVID-19 in different educational contexts in Iran and around the world (e.g. Fathi et al., 2011; Heidari, 2020). Based on researchers’ experience, the online courses which were initially designed for face-to-face classes were used as the medium of instruction in the distance mode. As to the quality of e-learning in the country, the findings indicate that e-learning courses are of good quality in some respects (Fathi et al., 2011; Rastegarpar & Gorjizadeh, 2012) but in general, these courses have not been considered to be of an optimal quality (Ghaedi et al., 2006; Rahmani, 2005; Saad Mohammadi et al., 2015). Perhaps, there are some factors that affect interaction and knowledge sharing among students and the poor knowledge sharing may hinder students’ reflectivity and as such the quality of online courses is negatively affected. As such, the researchers sought to investigate the following research questions:

1. What are the common knowledge sharing practices among English literature B.A students in online learning courses?
2. What are the English literature B.A students’ perceptions towards reflective thinking in their online learning courses?
3. Is there a significant relationship between individual, classroom, technological factors with English literature students’ knowledge sharing behavior?
4. Is there a significant relationship between knowledge sharing as a mediating variable and reflective thinking as a criterion variable?

## Method

### Design

In the present study, a quantitative and correlational design was adopted to address the research questions. Two questionnaires were used to collect the quantitative data, and then the data were analyzed using descriptive and inferential statistics.

### Participants and setting

The participants included 104 undergraduate B.A (Bachelor of Arts) student. They were studying English literature at the Department of English Language and Literature of a state university. Non-probability sampling technique (convenience/opportunity sampling) was employed to select the participants since it was impossible to draw random probability sampling. In addition, the participants were conveniently available to the researcher. They were composed of both male and female students (46.2% male, 53.8% female) and their ages ranged from 23 to 37. All students had the experience of two consecutive terms learning English literature via an online course after the inception of COVID-19 and because of university lockdown they were studying their courses totally online. The platform through which synchronous education is provided is LMS and the web-based software is currently employed in majority of Iranian state universities for accomplishing didactic tasks, assigning materials, initiating student interactions, making contacts with students, and assessing students. Among the challenges teachers face in the online courses are faculty’s adaptability struggles and students’ lack of instruction (Chahkandi, 2021).

### Data collection tools

#### Students’ online knowledge sharing behavior

To assess English literature students’ online knowledge sharing behavior the scale developed by Wangpipatwong (2009) was adopted. Since the original questionnaire was for face to face courses some modifications were made in the scale and items 13,14,17, and 18 were also replaced with those of Chennamaneni (2005) to suit online courses. Then, four experts in the field were required to pass their judgments on the items. There are 21 items in the scale using a five-point Likert ranging from “Strongly Disagree” to “Strongly Agree” as illustrated in Table 2.

The first and the second dimensions, willingness to share and ability to share are individual factors which indicate the individual’s knowledge sharing via verbal and/or written skill. The second dimension, classroom factors, involves instructor support and degree of communication. These two factors are related to the degrees the teacher conducts the class and provides the situation for communication and knowledge sharing. The third factor is technological which includes technology support and technology availability. This dimension is very important in knowledge sharing and particularly amid the Covid-19 crisis since technology can be regarded as the best and the only means to long-distance collaboration. Furthermore, as a facilitator, the use of technology can result in easier and more efficient knowledge sharing.

As reported by Wangpipatwong (2009), Cronbach’s alpha coefficient for the subscales were as follows: willingness to share (.797), ability to share (.790), instructor support (.716), degree of competition (.651), technology availability (.694), technology support (.823), and knowledge sharing (.728).

#### English literature students’ perceptions towards reflective thinking

A researcher-made survey based on the literature (Avarzamani & Farahian, 2019; Wang, 2014) was designed based on a 5‐point Likert scale to explore the participants’ attitudes and experiences towards reflective thinking. The scale included 15 items with a five-point Likert scale. After developing the first draft of the scale, two experts were asked to pass their judgments on the items. Based on their views, necessary revisions were made to the scale.

#### Reliability and validity of the instruments

In order to determine the validity of the two questionnaires, five faculty members, specializing in the subject of the article, were asked to express their opinions. Their comments indicated that the questionnaires had good content validity. Exploratory factor analysis (EFA) using principal component analysis, average variance extracted (AVE), and Cronbach’s alpha coefficient were used to estimate the validity and reliability of the questionnaire (Table 1).

**Table 1:** Validity and reliability of research variables using factor analysis and Cronbach's test

Constructs	Items	Loadings	AVE	Cronbach’s alpha	Cronbach’s alpha (Total)
Willingness to share	1. I am willing to discuss new ideas with my classmates in the online course	.953	.937	.896	.802
	2. I am willing to share knowledge that I acquire with my classmates in the online course	.952			
	3. I am willing to share course materials with my classmates in the online platform	.911			
Ability to share	1. I find it easy to put what I know into words	.915	.900	.826	
	2. I am confident in my ability to provide knowledge to my classmates	.914			
	3. I am confident that my knowledge sharing would increase the performance of my classmates	.881			
Instructor support	1. My instructor supports us in sharing knowledge with other classmates	.904	.854	.884	
	2. My instructor encourages us to discuss with other classmates	.879			
	3. My instructor gives us a reward, such as verbal praise and score, when sharing knowledge with other classmates	.878			
Degree of competition	1. I feel that my final grade is dependent on a great extent on the relative performance of my classmates	.851	.846	.737	
	2. I feel that my classmates have the potential to perform better than me	.803			
	3. I feel that my classmates are my competitors	.756			
Technology availability	1. I often experience difficulties in accessing the existing communication channel for sharing knowledge	.927	.909	.769	
	2. Tools and technology for sharing knowledge is available when it is needed	.866			
	3. Whenever I want to share knowledge there are some IT tools available for sharing knowledge	.742			
Technology support	1. I am satisfied with the overall quality of tools and technology for sharing knowledge in our university	.860	.805	.778	
	2. IT makes it easier for me to share knowledge with my classmates	.767			
	3. Tools and technology for sharing knowledge can be customized to fit individual needs	.753			
Knowledge sharing	1. I usually share with my classmates the new knowledge that I acquire	.887	.820	.849	
	2. I usually inform my classmates of what I am working on	.879			
	3. I always tell my classmates whatever I know when they ask me	.504			

The results of this test according to Table 1 showed the components of willingness to share (.897), Ability to share (.826), Instructor support (.884), degree of competition (.737), technology availability (.769), technology support (.778), knowledge sharing (.849) and the alpha coefficient of the seven subscales was .802. In addition, reflective thinking questionnaire (.749) showed an optimal reliability. Regarding the total variance, it should be stated that the first factor with a specific value of 4.431 alone could explain 21.101% of the total variance. The second factor, whose eigenvalue was equal to 3.377, was able to explain with 16.079% of the total variance. The third factor, with the eigenvalue of 2.883, accounted for 13.730% of the total variance. The fourth, whose eigenvalue was 2.467, explained 11.749% of the variance; the fifth factor, whose eigenvalue was 1.685, explained 8.024% of the total variance, and the sixth factor, which was 1.099 explained 5.233% of the total variance. In general, the seven extracted factors explained 82.658% of the total variance of knowledge sharing. This is an appropriate and acceptable level of variance.

### Data collection and data analysis procedure

The study was conducted during the spring term of the 2018–2019 academic year in Iran. The population of the study included English undergraduate literature students from six state universities. Since we did not have access to the participants because of the COVID-19 lockdown questionnaires were distributed via Google Forms. Of 195 English literature students, 104 uploaded the questionnaires. The data derived from Google Forms was uploaded to SPSS 23 and Amos 21 for analysis. It seemed necessary to run descriptive statistics to identify English literature students’ attitudes towards online learning and to find out the B.A students’ perceptions towards reflective thinking in their online learning courses. We also employed a Pearson’s correlation coefficient and path analysis to analyze the data and to generate a model.

**Fig. 1:**
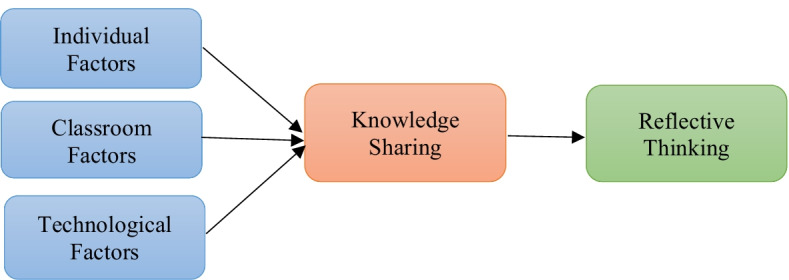
Conceptual model of research

Figure 1, shows the conceptual model of the research. In this model, independent variables include individual, classroom, and technological factors. The knowledge sharing variable is considered as the mediator dependent variable, and the reflection thinking variable is considered as the criterion dependent variable.

## Results

The first research question examined the common knowledge sharing practices among undergraduate students in online learning courses. The results of this question are shown in Table 2.

**Table 2:** Students’ online knowledge sharing behavior

Variable	Questions	Mean	Std. deviation	*t*	Sig. (2-tailed)
Willingness to share	1. I am willing to discuss new ideas with my classmates in the online course	3.24	.719	3.498	.001
	2. I am willing to share knowledge that I acquire with my classmates in the online course				
	3. I am willing to share course materials with my classmates in the online platform				
Ability to share	4. I find it easy to put what I know into words	3.39	.633	6.397	.000
	5. I am confident in my ability to provide knowledge to my classmates				
	6. I am confident that my knowledge sharing would increase the performance of my classmates				
Instructor support	7. My instructors support us in sharing knowledge with other classmates in the online course	2.81	.858	− 2.246	.027
	8. using the online platform, my instructor encourages us to discuss with other classmates				
	9. My instructors gives us a reward, such as verbal praise and score, when sharing knowledge with other classmates in the online courses				
Degree of competition	10. I feel that my final grade is dependent on a great extent on the relative performance of my classmates	3.49	.572	8.790	.000
	11. I feel that my classmates have the potential to perform better than me				
	12. I feel that my classmates are my competitors				
Technology availability	13. I often experience difficulties in accessing the existing communication channel for sharing knowledge	4.17	.500	23.983	.000
	14. Tools and technology for sharing knowledge is available when it is needed				
	15. whenever I want to share knowledge there are some IT tools available for sharing knowledge				
Technology support	16. IT makes it easier for me to share knowledge with my classmates	2.79	.455	− 4.664	.000
	17. Tools and technology for sharing knowledge can be customized to fit individual needs				
	18. I am satisfied with the overall quality of tools and technology for sharing knowledge in our university				
Knowledge sharing	19. I usually inform my classmates of what I am working on	2.75	.767	− 3.324	.001
	20. I usually share with my classmates the new knowledge that I acquire				
	21. I always tell my classmates whatever I know when they ask me				

According to Table 2, the results of the one-sample *t*-test show six subscales including willingness to share, ability to share, instructor support, degree of competition, technology availability, technology support, and knowledge sharing variable.

A. In the *willingness to share* subscale, the value of the one-sample *t*-test (*t* = 3.498) shows that with respect to the level of significance (Sig. = .001) there is a statistically significant difference between the sample population and the comparison value. Moreover, the value of the sample population (*m* = 3.24) is higher than the value of the comparison value (3).
B. In the *ability to share* subscale, the value of the one-sample *t*-test (*t* = 6.397) indicates that with respect to the level of significance (Sig. = .000) there is a statistically significant difference between the sample population and the comparison value. Moreover, based on the results, the value of the sample population value (*m* = 3.39) is higher than the value of the comparison value (3).
C. In the *instructor support* subscale, the value of the one-sample *t*-test (*t* = − 2.246) illustrates that with respect to the level of significance (Sig. = .027) there is a statistically significant difference between the sample population and the comparison value. In addition, as the results indicate, the value of the observed mean (*m* = 2.81) is less than the value of the hypothetical mean (3).
D. In the *degree of competition* subscale, the value of the one-sample *t*-test (*t*. = 8.790) shows that with respect to the level of significance (Sig. = .000) there is a statistically significant difference between the sample population and the comparison value. Moreover, according to the results, the value of the sample population value (*m* = 3.49) is higher than the value of the comparison value (3).
E. In the *technology availability* subscale, the value of the one-sample *t*-test (*t* = 8.790) reveals that with respect to the level of significance (Sig. = .000) there is a statistically significant difference between the sample population and the comparison value. Moreover, according to the results, the value of the sample population value (m = 4.17) is higher than the value of the comparison value (3).
F. In the *technology support* subscale, the value of the one-sample *t*-test (*t* = − 4.664) shows that with respect to the level of significance (Sig. = .000), there is a statistically significant difference between the sample population and the comparison value. The results reveal that the sample population value (*m* = 2.79) is less than the value of the comparison value (3).
G. In the *knowledge sharing* variable, the value of the one-sample *t*-test (*t* = − 3.324) shows that based on the level of significance (Sig. = .001) there is a statistically significant difference between the sample population and the comparison value. In addition, the value of the sample population value (*m* = 2.75) is higher than the value of the comparison value (3).

Overall, according to Table 2, the mean of the subscales willingness to share, ability to share, and degree of competition were higher than the comparison value (3), and the subscale of technology availability was more desirable than other subscales. In contrast, the mean of the two subscales instructor support, technology support, and the knowledge sharing variable were lower than the comparison value (3).

The second research question explored the English literature B.A student’ perceptions towards reflective thinking in their online learning courses. The results of this question are depicted in Table 3.

**Table 3:** English literature student’ perceptions towards reflective thinking in their online learning courses

Questions	Mean	Std. deviation	*t*	Sig. (2-tailed)
1. The online learning platform helped me to have a deeper knowledge of the course content	2.86	.360	− 3.694	.000
2. The online learning platform helped me analyze, synthesize and evaluate concepts and information in literary texts				
3. Using the online platform I have learned to compare my experiences to what I have read				
4. Using the online platform I have learned to discuss with others to deepen my understanding and explore a range of perspectives				
5. The online learning platform helped me interpret and value ideas expressed in literary texts				
6. The online platform helped me question the ideas or considering them in depth				
7. The online learning platform enabled me to reflect on what I learn				
8. The online platform helped me reflect on the literary texts I write				
9. The online platform increased my performance in writing for and against certain positions and ideas in literature courses				
10. The online platform helped me identify meaningful components of literary texts				
11. The online platform helped recall, and retrieve the course content				
12. Collaboration through the online platform helped me consider a range of information derived from many different sources				
13. The online platform helped me to evaluate ideas from different perspectives				
14. The online platform helped me shift from superficial/descriptive responses to critical consideration of issues				
15. Using The online platform helped me to take a stand when reading literary texts				

According to Table 3, the results of the one-sample *t*-test (*P* = .000 < 0.05) illustrate that there is a statistically significant difference between the actual or observed mean and the hypothetical mean. Moreover, the value of the sample population value (*m*. = 2.86) is less than the value of the comparison value (3). Thus, it can be inferred that the reflective thinking subscale is not at an acceptable level.

The third research question inquired whether there is a significant relationship between individual, classroom, and technological factors with English literature students’ knowledge sharing behavior. To answer this question, first, the subscales and then the main factors are examined. The results of this question are shown in Table 4.

**Table 4:** Relationship between willingness to share, ability to share, instructor support, degree of competition, technology availability, and technology support with knowledge sharing

	Knowledge sharing	Willingness to share	Ability to share	Instructor support	Degree of competition	Technology availability	Technology support
Knowledge sharing	1						
Willingness to share	.536**	1					
Ability to share	.542**	.290**	1				
Instructor support	.433**	.417**	.459**	1			
Degree of competition	.516**	.348**	.599**	.310**	1		
Technology availability	.557**	.473**	.374**	.285**	.447**	1	
Technology support	.709**	.450**	.391**	.485**	.251*	.372**	1

*Correlation is significant at the 0.05 level (2-tailed), **Correlation is significant at the 0.01 level (2-tailed)

According to Table 4, the results of the Pearson correlation coefficient showed that based on the obtained significance level (Sig. = .000) there is a significant relationship between the variables of willingness to share, ability to share, instructor support, degree of competition, technology availability, and technology support with knowledge sharing. In terms of the intensity of correlation, the variables of willingness to share (*r* = .536), ability to share (*r* = .542), instructor support (*r* = .433), degree of competition (r = .516), technology availability (*r* = .557), and technology support (*r* = .709) were almost moderate to strong.

As the next step, it was necessary to examine the relationship between these three components (individual factors, classroom factors, technological factors) and knowledge sharing. As such, the Pearson correlation coefficient was employed. The results of this test are shown in Table 5.

**Table 5:** The relationship between individual, class, and technological factors with knowledge sharing

	Knowledge sharing	Individual factors	Classroom factors	Technological factors
Knowledge sharing	1			
Individual factors	.671**	1		
Classroom factors	.580**	.695**	1	
Technological factors	.756**	.635**	.547**	1

**Correlation is significant at the 0.01 level (2-tailed)

As Table 5, illustrates, a significant level of (Sig. = .000) has been obtained, so it can be inferred that there is a significant correlation between individual factors, classroom factors, and technological factors with knowledge sharing. In terms of intensity, the correlation was strong. Therefore, the research H_1_ is confirmed, but the H_0_ is rejected.

The fourth question of the study explored whether there is a significant relationship between knowledge sharing as a mediating variable and reflective thinking as a criterion variable. Pearson correlation coefficient was used to investigate this question. The results are shown in Table 6.

**Table 6:** Relationship between knowledge sharing and critical thinking

variable	*N*	Pearson correlation
The relationship between knowledge sharing and reflective thinking	104	.623**

**Correlation is significant at the 0.01 level (2-tailed)

According to Table 6, there is a significant relationship between knowledge sharing and reflective thinking (Sig. = .000). Hence, in terms of intensity, the correlation (*r* = .623) between the two variables is strong. Therefore, the research *H*_1_ was confirmed and the *H*_0_ was rejected.

### Model fit

**Fig. 2:**
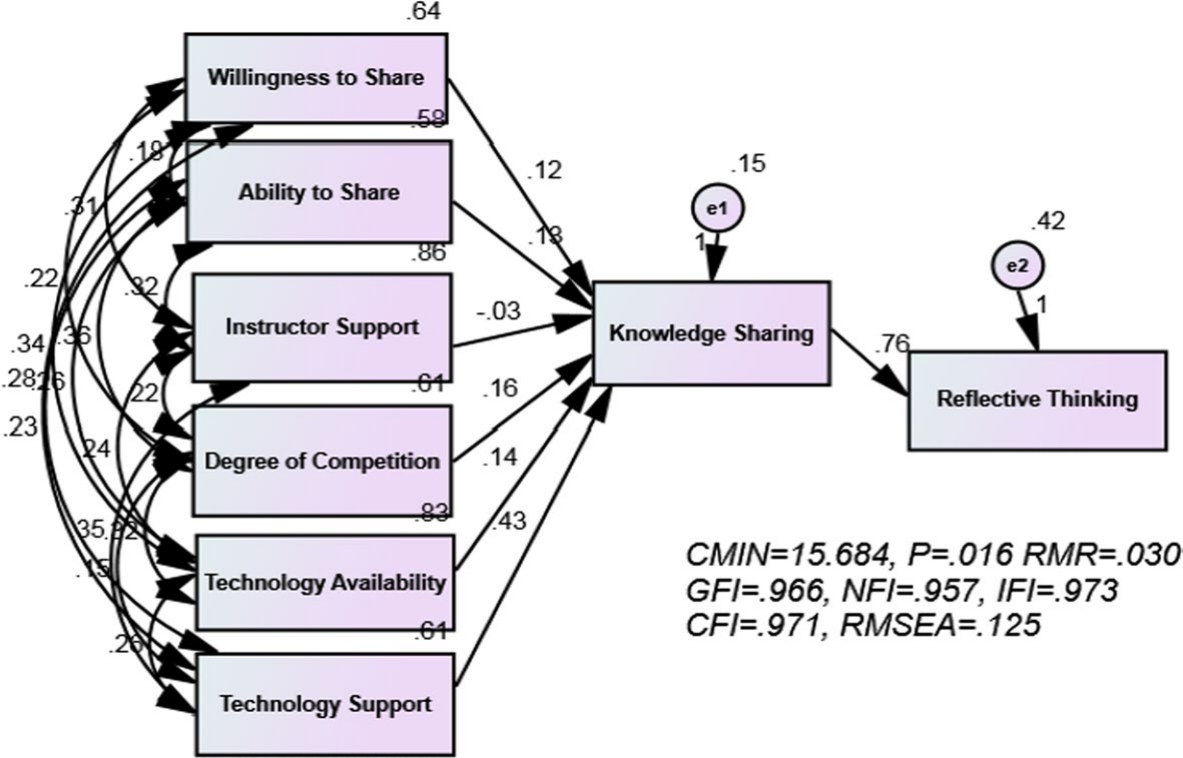
The final model A with six subscales

**Fig. 3:**
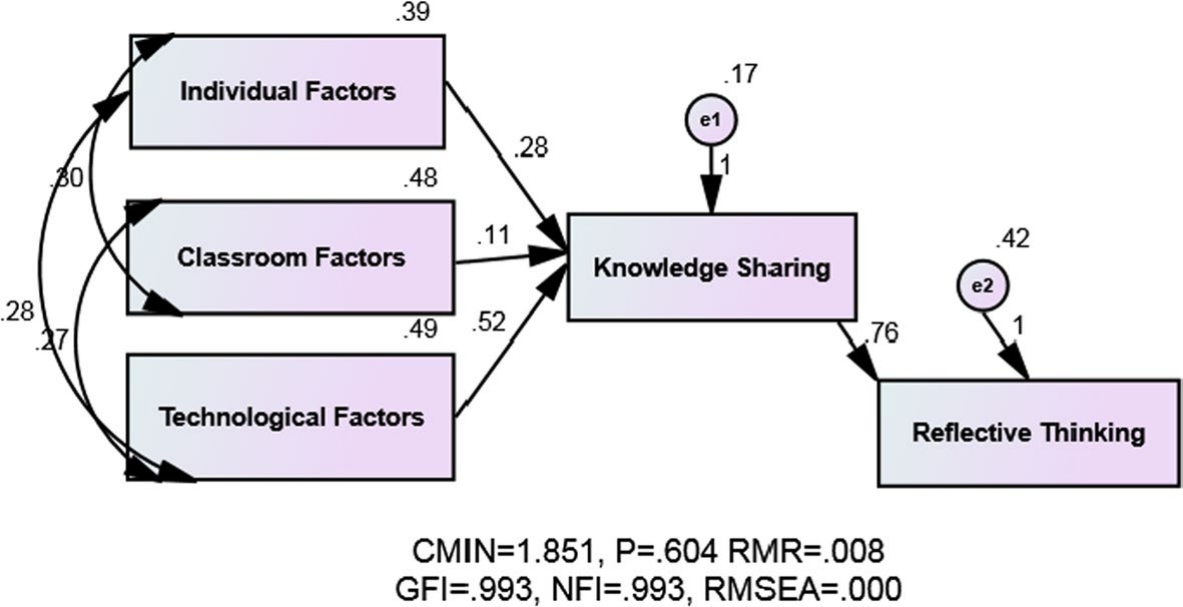
The final model B with three main components

Finally, we inquired whether the proposed model has a good fit. To answer this question, path analysis was used. Hence, the present study sought to determine the best possible path between the variables of individual factors (willingness to share, and ability to share), classroom factors (instructor support, and degree of competition), and technological factors (technology availability, and technology support). The results of the path analysis are shown in Figs. 2 and 3 (Table 7).

**Table 7:** Index of fix indices model

Index name	(CMIN) (χ^2^)	(CMIN/DF)&P	NFI	CFI	GFI	AGFI	RMR	RMSEA	PNFI	PCFI
Fitting adequacy value	15.684	2.614 (*P* = .016)	.957	.971	.996	.795	.030	.125	.205	.208

Based on the statistics of chi-squire (CMIN) (× 2) is 15.684 and relative (CMIN/DF) is 2.614, with *P* = .016 > 0.05. It can be inferred that there is an adjustment between the suggested model and the observed data. The factors GFI = .996, AGFI = .795, CFI = .971, NFI = 957 were equal or close to one. It can be inferred that the model enjoyed a good fit. The RMR values also indicated a good fit. The results of absolute, adaptive and parsimonious indices showed there is a good fit in the communication model between the willingness to share, ability to share, instructor support, degree of competition, technology availability, and technology support as the predictive variable with the knowledge sharing as the mediator dependent variable, and reflective thinking as the criterion dependent variable. In general, the indicators introduced in the study showed that although the second root index (RMSEA) of the mean squares were not satisfactory. Different researchers have reported different acceptable values for RMSEA index. For example, Browne and Cudeck (1993) indicated that the RMSEA population parameter values of about 0.05 or less indicate a close fit of the model, and values of about 0.08 or less indicate a reasonable approximate error. Hu and Bentler (1999) have argued that RMSEA values less than 0.06 is acceptable. McCallum et al. (1996) identified 0.01, 0.05, and 0.08, respectively, as excellent, good, and moderate fitness. However, this index alone cannot be the only indicator of the fit of a model. Because some researchers believe that the degree of freedom affects the RMSEA index. As an example, Chen et al. (2008) state that sample size clearly affects the performance of the RMSEA. However, with simpler models with few degrees of freedom RMSEA has serious problems. This is especially the case with simple path models and simple CFAs, which often have few degrees of freedom. In such cases, even when the model fits the data well the RMSEA may wrongly indicate a poor fit (Kenny et al., 2015).

In addition to presenting the subscales of individual factors, classroom, and technology, here we present a model based on three main factors, namely individual factors, classroom factors, and technological factors as independent variables on knowledge sharing and reflective thinking.

Based on the data given in the Table 8, the chi-squire (CMIN) (× 2) is 1.851 and relative (CMIN/DF) is 0.617, with df = 3 and *P* = .604 > 0.05. It can be inferred that there is an adjustment between the suggested model and the observed data. The factors GFI = .993, AGFI = .965, CFI = .996, NFI = .993, were equal or close to one. It can be inferred that the model enjoyed a good fit. Furthermore, the RMSEA value of .000 < .000, and RMR value of .008 are indicative of the good fit. The results of absolute, adaptive, and parsimonious indices showed that there is a good fit in the communication model between the individual, classroom, and technological factors while the predictive variable was knowledge sharing as the mediator dependent variable, and reflective thinking as the criterion dependent variable. Furthermore, there is a linear relationship between the intervening variable and the latent variables. It can also be inferred that there is a linear relationship between variables and the latent variables indicating that the model enjoys a good fit.

**Table 8:** Index of fix indices model

Index name	(CMIN) (χ^2^)	(CMIN/DF)&P	NFI	CFI	GFI	AGFI	RMR	RMSEA	PNFI	PCFI
Fitting adequacy value	1.851	.617 (*P* = .604)	.993	.996	.993	.965	.008	.000	.298	.300

To calculate the mediating effects, a Bootstrap approach was used to assess the direct and indirect effects. The effect of each of the predictor variables, including individual factors, classroom factors, and technological factors, directly and indirectly on the intermediate dependent variable (Knowledge sharing) and reflective thinking was estimated. The results are shown in Table 9.

**Table 9:** Standardized loadings for direct, indirect, and total effects

Predictor	Criterion	Direct effects	Indirect effect	Total effect
Individual factors	Knowledge sharing	.255	.000	.255
Classroom factors	Knowledge sharing	.112	.000	.112
Technological factors	Knowledge sharing	.533	.000	.533
Knowledge sharing	Reflective thinking	.623	.000	.623

According to Table 9, knowledge sharing plays a mediating role between predictor variables and reflective thinking.

## Discussion

The results of the first research questions revealed that English literature students’ knowledge sharing behavior is not at an optimal level. The results are consistent with the meta-analysis conducted by Dokhtesmati and Ghorbani Bousari (2013) who investigated the condition of factors affecting knowledge sharing in Iranian universities according to three criteria of human, organizational, information technological factors are not in good condition. As they explain, knowledge sharing does not have an acceptable status in the Iranian educational context. Furthermore, as they maintain, Iranian students do not have a positive attitude toward knowledge sharing. In line with the findings, Abbaszadeh and qasemzadeh (2018) also reported that the level of knowledge sharing in Iranian universities is not acceptable. In the same vein, Dokhtesmati and Ghorbani Bousari (2013) concluded that many negative factors affect knowledge sharing in Iranian academic institutions. The finding of the present study regarding the level of knowledge sharing might be ascribed to the emergency condition in the country where thousands of English teachers are relying on online courses as the only way to teach while some have little experience to handle the situation effectively and are prompted to do so. Perhaps, the situation has led the students have little opportunity and/or inclination to embark on online interaction with peers and their teachers. Since knowledge sharing among university students has been recognized to be important universities should develop and structure the curriculum in a way that engage students in collaborative learning and allow them to reflect and learn.

Based on the results of the second research question, the English literature students believed that literature courses through online courses do not enhance their reflectivity. No study, to the researchers’ knowledge, has investigated Iranian university students’ attitudes towards reflective thinking in the Iranian context especially in online courses. In a study which is partially in tandem with the present study, Avarzamani and Farahian (2019) researched Iranian students’ reflectivity in writing and found that Iranian EFL writers have noticeable weaknesses in implementing reflective thinking while writing and they are mostly involved in lower levels of reflection. As to the efficacy of online courses in enhancing students’ reflectivity, contrary to the present study, a few studies have reported that employing online education can promote the students’ creativity (Chang, 2012; Jang, 2009), their active engagement with the course and promotes higher-order thinking (Al Fadda & Osman, 2020).

Although it has been claimed that online discussions can effectively coach and develop reflective learning (MacKnight, 2000), the findings of the study come as no surprise, since Iranian university courses are fairly exclusively confined to teacher-oriented approaches and Iranian English teachers have not been trained to incorporate reflectivity in their courses (Farahian, et al., 2021); like many Asian countries (see Rear, 2017) English teaching faculties in Iran have often the inclination to give the utmost priority to learning the content of the course and language proficiency rather than cultivating higher-order thinking skills (Janebi Enayat et al., 2015) and so like any other collectivist cultures, learning activities are limited to knowledge and comprehension level rather than higher-order thinking. On top of that, Iranian teachers are not willing to give up their authority (Safari & Pourhashemi, 2012).

The third research question inquired whether there is a significant relationship between individual, classroom, technological factors and their subcomponents with English literature students’ knowledge sharing behavior. The results showed a significant relationship between all variables. In a similar study, Wangpipatwong (2009) reported that among all factors only three, namely, technology support, ability to share, and degree of competition, had a significant impact on knowledge sharing. While technology support and ability to share positively impacted knowledge sharing, degree of competition negatively affected the students’ knowledge sharing. The findings can also lend support to a similar study conducted by Nisar ul Haq and Haque (2018) who found that trust, attitude and information and communication technology (ICT) use are the key factors in order to improve knowledge students’ knowledge sharing. Jantavongso and Nuansomsri (2019) also concluded that "intention to share", "technological", "individual", and "classroom" factors influenced knowledge sharing among ICT undergraduate students. This indicates that raising English literature teachers’ awareness of technology assets, human resources, and classroom factors is necessary in order to achieve an acceptable level of knowledge sharing among students. Accordingly, it is necessary to take into consideration both negative factors hindering interaction and knowledge sharing and at the same time the positive factors that promote knowledge sharing in online courses.

The fourth research question explored whether there is a significant relationship between knowledge sharing as a mediating variable and reflective thinking as a criterion variable. The findings indicated that there is a significant relationship between knowledge sharing and reflective thinking. This gains support from the literature (Alblehai & Umar, 2016; Lipman, 1991; Vygotsky, 1978) which considers social interaction and scaffolding as affecting students’ higher-order thinking and mental development. The results indicate that although there is the claim that web-based technology encourages both problem-solving skills (Zarei et al., 2019) and offers ‘interactive educational experiences’ as well as social interaction (Newman et al., 1997; Xia, 2013, p.104), the participants’ perceptions towards reflective thinking in their online learning courses were not positive. This supports the suggestion that although, as discussed in the literature (see McLoughlin & Mynard, 2009), online discussion forum is a technology that develops collaborative meaning-making and fosters higher-order thinking, they “do not necessarily support higher levels of learning” (Garrison & Kanuka, 2004). It appears that, what has deteriorated the underdevelopment of reflectivity is the hasty integration of e-learning into the educational context under the menace of COVID-19 where the conditions have not been prepared for such an undertaking. As the consequence, students have not had the opportunity and training to interact with peers, share the contents, and helps with building connections. This may cast light on the issue that although technology helps develop higher order thinking skills (Kwangmuang et al., 2021; Yaniawati, 2013) if proper training is not provided for teachers as in-service training courses, they may consider the online forum as the place for conventional lectures as the traditional classroom and hence deprive their students of enhancing thinking skills.

Overall, the results indicated that the proposed model is acceptable and that the final model had an acceptable fit with the empirical data. One possible explanation for the findings could be that active learner participation in a social context promotes deep learning. In this regard, Lipman claims that developing a ‘‘community of enquiry’’ is necessary for the development of higher-order thinking within the individual (Lipman, 1991). Such communities of inquiry can be found in group learning. Perhaps, due to the weak technological support, and instructor support the technology did not act at its optimal level as a medium.

Based on the researchers’ experience, the knowledge sharing process in the Iranian higher education online system lasts for a short time. The possible explanation, as the findings indicated, is related to the technological support. Technological factors, like lack of adoption of the systems or poor user-friendliness of information systems can have severe consequences on hindering users from participating in online systems (Au Leung & Law, 2013; Tabatabaie, 2010; Yeung & Law, 2006). Additionally, low speed of the Internet, cost of the Internet connection, and weakness of the Internet networks have also aggravated the problem in the country.

A second explanation which parallels our findings is the lack of sufficient instructors’ support. As a facilitator, the e-learning teacher should be familiar with educational goals and identify the right type of learning activities (Khorasani et al., 2017). It is likely that the instructors who are accustomed to using didactic (i.e., lecturing) approaches to teaching may continue using the same approach while teaching in online platforms (Roblyer & McKenzie, 2000). Furthermore, research also shows that Iranian teachers do not have sufficient skills in using virtual education courses (Daneshvar & Mehr Mohammadi, 2013). Since online teachers experience challenges different from those of physical courses (see Baran et al., 2011) and have three major roles, namely, pedagogical, managerial and social (Coppola et al., 2002).

### Pedagogical implications

Although online courses seem to be the best substitute for face-to-face physical courses, some conditions should be met in designing online courses. As such, several practical implications emerge from the findings. First, raising teachers' awareness regarding the role of individual factors such as students' personality and attitude in students' interaction seems essential. It is likely that in online courses, personality factors have a stronger effect on students' interpersonal interactions and that can impact the quality and quantity of knowledge sharing. Future research can shed light on the issue. Secondly, it seems that the time span for faculty teaching has decreased in online participation and unlike 1.30 face-to-face class hours most classes run less than the allocated time. Accordingly, less time is given to students to have interactions during the course. This could be compensated by allocating some time by the instructors to students to have access to the instructors and other students via the social media under teachers' supervision. This may result in more knowledge sharing in online courses. Thirdly, size of the classes and the number of students who take part in online courses, as classroom factors, is important since there is a fear that overcrowded courses does not provide opportunities for all students to have optimal interactions with peers and their instructor. Finally, as suggested by Barbour and Plough (2009) and Hawkins et al. (2011), learner–teacher interaction can be maximized through class discussions, feedback to students, emails, and social media.

## Conclusion

Despite the benefits, online learning has resulted in student’ less personal relationship with their teachers and classmates and it seems that there is still a long way to achieve effective knowledge sharing in online courses. As such, it appears that training for professors and students, improving technical design planning of instructional and material design are required.

For an effective knowledge sharing to take place teachers’ awareness regarding the differences between online and physical education should be raised and through courses and workshops they should be trained to employ strategies of promoting learners’ interaction in their classrooms (Kasani et al., 2020). At the same time, information technology used in the university should be easy to use, easy to access, integrative, and searchable (Bartol & Srivastava, 2002).

The major limitation in the present study is the participants’ self-report. Perhaps, participants may inflate the real information and do not provide their real attitude. This endangers the validity of the findings. Accordingly, in further studies, qualitative research methods can be adopted. Another limitation is the characteristics of the population of the study. Although the main focus of the study was on English literature students, further studies can include students from other fields of English such as TEFL (teaching English as a foreign language), translation, and linguistics. Furthermore, we did not consider the role of the atmosphere of the courses in the model. Future studies can investigate if positive attitude among students can contribute to a different result in the model.

## Data Availability

Not applicable.

## Abbreviation

EFL: English as a foreign language
